# Can the household clean energy transition ameliorate health inequality? Evidence from China

**DOI:** 10.3389/fpubh.2024.1348234

**Published:** 2024-03-20

**Authors:** Lili Wu, Qin Liu, Lin Li

**Affiliations:** School of Economics and Management, China University of Petroleum, Beijing, China

**Keywords:** household energy transition, health effects, health inequality, CFPS, China

## Abstract

China is actively encouraging households to replace traditional solid fuels with clean energy. Based on the Chinese Families Panel Survey (CFPS) data, this paper uses propensity scores matching with the difference-in-differences model to examine the impact of clean energy in the household sector on residents’ health status, and whether such an energy transition promotes health equity by favoring relatively disadvantaged social groups. The results show that: (1) The use of cleaner cooking fuels can significantly improve residents’ health status; (2) The older adult and women have higher health returns from the clean energy transition, demonstrating that, from the perspective of age and gender, the energy transition contributes to the promotion of health equity; (3) The clean energy transition has a lower or insignificant health impact on residents who cannot easily obtain clean energy or replace non-clean energy at an affordable price. Most of these individuals live in low-income, energy-poor, or rural households. Thus, the energy transition exacerbates health inequalities. This paper suggests that to reduce the cost of using clean energy and help address key issues in health inequality, Chinese government efforts should focus on improving the affordability, accessibility, and reliability of clean energy.

## 1 Introduction

The Research Report on Household Energy Consumption in China (2016) indicates that the proportion of traditional biomass energy, such as firewood and straw, in Chinese household energy consumption is as high as 61%. Harmful substances generated by incomplete combustion of solid fuels are one of the important sources of indoor air pollution, which has become a leading health threat (1) and contributor to induced diseases, including chronic obstructive pulmonary disease, lung cancer, and infant mortality (2, 3). Since the 13th-5-Year-Plan period, China has promoted the use of clean energy on a large scale. “Coal to electricity” and “coal to gas” policies, as well as measures like the development of new energy sources, have encouraged residents to replace solid fuels with clean energy. In 2015, the Chinese central government implemented a new environmental protection law that is the strictest in China’s history (4). To better implement this new law, China’s central government empowers local governments to formulate corresponding plans based on the actual conditions in their regions and incorporates environmental protection objectives into the assessment system of local government officers, urging local governments to pay closer attention to environmental governance. At the same time, to reduce environmental pollution, China’s local environmental regulators have placed restrictions on the use of solid fuels by households. Through policy support and law enforcement, energy consumption structures in China’s household sector have been largely transformed from traditional to modern sources. This transition has significantly reduced the emissions of harmful gasses and produced broad health benefits (5).

Recent studies show that the transition from polluting to clean household energy consumption can improve residents’ health status, but studies of its impact on the health profile of different population groups remain limited. Many studies based on Chinese rural data show that clean energy adoption can efficiently improve the health of vulnerable groups who suffered severe environmental health hazards caused by solid fuel. For example, where construction of older adult care facilities lags in rural areas, the older adult spend less time outdoors and more time cooped up in houses, in which they are exposed to environmental hazards produced by the use of solid fuels. Women spend a greater proportion of their time in the household and bear much of the responsibility for cooking, cleaning, and childcare. Therefore, the older adult and women are particularly vulnerable to diseases caused by indoor air pollution from solid fuels, and the use of clean energy could notably improve their health (6–13), indicating that the energy transition can promote health equity. These studies are all based on data from rural China, but Chinese cities have also experienced the process of replacing clean energy. China Urban Household Survey data shows that from 2002 to 2015, the coal consumption of urban households in China decreased by 78.2%, while the consumption of electricity and natural gas increased by 121.9 and 88.1%, respectively. However, to date, no studies have used Chinese data to examine how the household energy clean transition has impacted residents’ health in urban and rural populations. We posit that analyzing the energy transition’s impact on these groups can help explain differences in health outcomes.

Furthermore, although China has achieved full electricity coverage through its gradual advancement of the power grid, the construction of other components of energy infrastructure, such as gas pipelines, has lagged behind. Consequently, many residents have been unable to meet their daily energy requirements. According to the 2015 China Household Energy Consumption Research Report, the *per capita* energy consumption of Chinese households is less than 50% of that of developed countries. The problem of energy poverty among residents is serious. This problem in developing countries like China is manifested not only in a lack of energy availability but also in energy’s non-affordability (14). Although promoting the use of clean energy and tightening regulations for solid fuel use can reduce their adverse impacts on the environment and people’s health, these steps can also burden households by forcing them to use costly clean energy, particularly in the case of low-income and rural households that lack access to clean energy (15). Will the health status of these vulnerable groups be affected by their inability to access the clean energy they want and need? Or will opportunities for improving health outcomes be squeezed out by clean energy expenditures? Although China recognizes the need to implement the “precision poverty alleviation” policy and alleviate energy poverty to improve the health and welfare of its residents, research to date that has examined the impact of the clean energy transition on the health of energy-poor groups is inadequate, and so the differences in how the clean energy transition affects the health improvement of energy-poor and income-poor groups remain poorly understood.

Additionally, the current research on household energy consumption and health uses mostly macro data. The studies that use microdata mostly examine cross-sectional data (16, 17), which are disadvantageous in the respect that they capture only snapshots in time of energy consumption patterns within populations; they cannot easily control for variation in the unobserved characteristics in populations that influence the developmental indicators of interest, such as health status. Panel data, which capture changes in the same set of individuals over time, can isolate the health and developmental effects of the clean energy transition on individuals while controlling for factors that vary across households. Recently, several studies have used panel datasets collected from two waves of surveys to identify changes in the health levels of rural households before and after improvements in the fuel structure (13, 18). Although these studies compensate for shortcomings in the cross-sectional data, they can cause errors because their sample time range is limited.

To bridge the research gaps described above, this paper proposes three analytical innovations. First, we select a new variable, the energy transition in Chinese households (rural and urban), as the entry point, which shifts the focus of analysis from examining the health risks caused by households’ energy consumption structure to determining whether optimizing that energy structure improves residents’ health. Second, to measure the health equity outcomes of the energy transition, we carry out a special examination of whether the energy transition benefits vulnerable social groups. Defining group “vulnerability” in terms of socioeconomic and energy-poor status, we carry out heterogeneity tests to further measure differences in the energy transition’s health effects. Finally, we use the China Family Panel Studies (CFPS) dataset collected in 2012, 2014, 2016, and 2018 to quantify the progress of China’s clean energy transition as well as its health benefits and its impact on health-related inequality at the household level.

The rest of this paper is organized as follows: Section 2 conducts a theoretical analysis and proposes research hypotheses. Section 3 describes the methodology, data sources, and variable selection. Section 4 uses descriptive statistics and basic regression analysis to verify our research hypotheses. Heterogeneity analysis is conducted in this part. Section 5 summarizes the research conclusions and proposes corresponding policy recommendations.

## 2 Theoretical analysis and research hypotheses

The health effect of the household energy transition refers to the use of clean energy as a substitute for solid fuels in the household sector—a substitution that improves residents’ health status. To illustrate the impact of the energy transition on residents’ health status, we refer to research by Song and Li (13) and construct a health utility function. In the function $U\left(i,m\right)$ , i represents the residents’ income and m represents their health consumption (medical expenditure). Residents are given only two states: healthy and sick. The probability that residents are healthy is p and the corresponding utility function is $U^{h}$ , under which the medical expenditure m=0 and the marginal utility of health consumption is constant zero. When a resident is sick, the probability is $\left(1-p\right)$ and the utility function is $U^{s}$ . The problem of maximizing health utility is as follows:


$$\underset{m}{\;\max}pU^{h}\left(i,0\right)+\left(1-p\right)U^{s}\left(i-m,m\right)$$

(1) s.t.m≤i

The optimal health consumption m * should meet the first-order condition:


(2) $$U_{1}^{s^{\prime }}\left(i-m\right)=U_{2}^{s^{\prime }}\left(m\right)$$

The equation (2) indicates that under the condition of being sick, the marginal utility of medical expenditure is equal to that of the remaining consumption expenditure, and the health utility of residents is affected by income i through changes in total income and total expenditure.

To simplify the analysis, the health utility function is set to quasi-linear form, as shown in the following equation:


(3) $$U\left(i-m\right)=i-m+H\left(m\right)$$

where $H\left(m\right)$ is an increasing function. According to equations (2, 3), in the state of being sick, the optimal healthy consumption $m^{*}$ satisfies $H^{\prime }\left(m^{*}\right)=1$ .

We assume that residents invest e in the clean energy transition to improve their living environment; thus, the health probability p is not completely exogenous and can be considered a function of energy investment—i.e., $p=f\left(e\right)$ . As energy investment increases, the probability that residents are healthy also increases, meeting $f^{\prime }\left(e\right)>0,f^{\prime \prime }\left(e\right)<0,\lim_{e\to \infty }f^{\prime }\left(e\right)\to 0$ . Therefore, Hypothesis 1 is proposed.

*H1*: The clean energy transition improves residents’ health status. When clean energy replaces solid fuels in the household sector the probability that residents will be healthy increases.

Increasing energy investment can improve residents’ health status. On the other hand, diminishing marginal outputs is also a factor in the field of health It can be intuitively inferred that among residents who have a higher-than-average health stock, the improvement effect that the household energy consumption transition has on health may be small, while the improvement effect it has on the health of relatively disadvantaged groups may be great. In reality, compared to younger people and male residents, older people and women are more likely to have poor health and suffer from the environmental hazards associated with solid fuel use. Unlike young and middle-aged residents, older people’s economic resources are greatly affected by the support of their children. Thus, because of limited resources, the older adults may rely more on traditional solid fuels and perhaps are less likely than younger residents to install range hoods and adequately ventilate their homes. Moreover, within the household division of labor, women do more cooking than men, and in households with limited resources, women are unequally exposed to coal and firewood fumes, which compromise their health. Therefore, in households where solid fuel is the main energy source, older people and women are likely to suffer the worst health consequences. In summary, the clean energy transition may be particularly beneficial to the health of relatively disadvantaged social groups. Therefore, Hypothesis 2 is proposed.

*H2*: In different populations, the health effects of the household clean energy transition differ. For example, the older adults and women benefit inordinately from the transition, which narrows the health gap.

In addition, as mentioned above, the health probability p changes with energy investment. In this case, the problem of maximizing residents’ health utility is


$$\underset{m}{\max\;}i-e-\left(1-p\right)m+H\left(m\right)$$

(4) $$s.t.\;e+\left(1-p\right)\;m\leq i$$

Now the optimal health consumption is $m^{**}$ . According to the first-order conditions, $m^{**}=H^{\prime }\left(p\right)$ . When p <1, $m^{**}<m^{*}$ . In other words, the energy transition leads to an increase in household energy consumption and a decrease in household health consumption. Limiting residents’ access to non-clean energy would force households that depend on non-clean energy to turn to high-cost clean energy—a step that leads to the problem of energy affordability. The energy transition is a burden in low-income households, and its costs can squeeze household expenditures that otherwise during the same period could be used to improve health. Something similar happens in energy-poor households due to their originally high energy burden: expenditure-based energy poverty increases as these households transition away from traditional energy. Because low-income and energy-poor households are mostly concentrated in rural areas, residents there may be deeply troubled by the crowding-out effect, resulting in a decrease in health utility that is likely higher than the increase caused by the energy transition. Based on the above analysis, we propose Hypothesis 3.

*H3*: Because the energy costs associated with the clean energy transition are high, large amounts of other consumption are squeezed out, and residents from low-income, energy-poor, and rural households (i.e., disadvantaged households) tend to experience health effects that are more modest than those experienced in non-disadvantaged households. The result is an economically generated health gap.

## 3 Methodology and data

### 3.1 Model setting

#### 3.1.1 Did method

In this paper, we examine whether and to what extent the clean energy transition in the household sector affects residents’ health in China. We examine the difference in the health of two groups of people: those whose households have undergone an energy transition; and those whose households have not. A difference in difference (DID) model is constructed to explicitly examine the causal effect of upgrading household energy consumption on residents’ health and its magnitude. DID utilizes both intra-group and inter-group variation of variables, which could improve the accuracy of estimation results. Moreover, DID allows for the presence of unobserved factors, and through double difference, it eliminates the impact of potentially unobservable features of individuals and the impact of common trends experienced by the two groups. Therefore, we use the DID method for estimation:


(5) $${\mathrm{Health}}_{\mathrm{it}}=\beta_{0}+\beta_{1}{\mathrm{Tran}}_{\mathrm{it}}+\sum \gamma_{j}X_{\mathrm{it}}+T_{t}+P_{i}+\epsilon_{\mathrm{it}}$$

where ${\mathrm{Tran}}_{\mathrm{it}}$ is the key explanatory variable, which is a difference in difference term obtained from ${\mathrm{Treat}}_{i}\times {\mathrm{Time}}_{\mathrm{it}}$ ,representing the clean energy transition in a household. ${\mathrm{Treat}}_{i}$ is a treated/control group dummy variable that equals 1 when the respondent belongs to the energy transition group (i.e., the treated group), and is 0 otherwise. ${\mathrm{Time}}_{\mathrm{it}}$ represents an event dummy variable. In the treated group, if individual i has replaced solid fuel with clean energy at time t , the value of ${\mathrm{Time}}_{\mathrm{it}}$ equals 1, and if no energy transition has occurred at time t , the value is 0. For control group individuals, all values of ${\mathrm{Time}}_{\mathrm{it}}$ are 0. $X_{\mathrm{it}}$ represents the control variable. $T_{t}$ and $P_{i}$ are time and province dummies, and they are used to control for time-fixed effects and province-fixed effects, respectively.

#### 3.1.2 PSM-Did method

To better reduce the selection bias, and on the basis of the DID model, we employ the propensity score-matching difference in difference method (PSMDID). This method not only makes use of the advantages of the DID method; through “propensity score matching,” it can also effectively control the difference in observable characteristics between the transition group and the non-transition group, thereby satisfying as much as possible the “conditional independence hypothesis.” Comparing nearest neighbor matching and caliper matching, we find that the 1:1 caliper matching method is best. A balance test is then conducted on the transition group and the non-transition group. The results show that the covariates of the two groups no longer have significant differences in the base period after matching, which indicates that the matching quality is higher. Following the balance test, this paper estimates the average treatment effect (ATT) of the household energy transition. The symbolic performance and significance of the regression coefficient are consistent, indicating that the treated effect obtained is relatively robust.

### 3.2 Data explanation

#### 3.2.1 Data source

We collect the household data from China Family Panel Studies (CFPS) conducted by the Institute of Social Science Survey of Peking University. This nationwide, large-scale, and multidisciplinary social longitudinal survey began in 2010 with a baseline survey that covered 25 provinces, cities, and autonomous regions across the country. The CFPS survey consists of four questionnaires: a community questionnaire, a family questionnaire, an adult questionnaire, and a child questionnaire. Each covers a broad range of economic and demographic characteristics at the household level. For this study, the questions about living conditions in the CFPS household questionnaire provide relevant information about the energy transition, household income, household size, and housing property rights, while the adult questionnaire covers basic information, such as residents’ gender, age, and education as well as residents’ self-assessment of their health status and behavior.

Our sample uses data from the 2012, 2014, 2016, and 2018 surveys; these range from July 2012 through April 2019 and cover 31 out of 34 provincial administrative regions. We select household and adult databases and merge them according to household ID and individual ID. We retain in the database those individuals who were continuously tracked during the four periods. After data cleaning, 34,467 cases are retained for statistical analysis.

#### 3.2.2 Variable selection

1. The dependent variable: residents’ self-rated health. Self-rated health is the respondents’ overall evaluation of their own health status. Although it is subjective, it is a comprehensive judgment of health. Self-rated health is not only highly relevant to objective indicators such as mortality and morbidity; it also contains information about disease severity, disease history, and health status stability. In other words, it can reflect respondents’ health status (15), and, consequently, it has been widely applied in the field of health economics. Considering two health statuses in our theoretical model and referring to the research of Fang and Lu (19) and Idler and Benyamini (20), we convert self-rated health into a binary dummy variable (1 = Very healthy, healthy, and relatively healthy; 0 = unhealthy and average).
2. Core independent variables: the clean energy transition. The construction of this dummy variable mainly comes from the item “whether the resident’s household has shifted from using firewood and coal as the main cooking fuel to using clean energy such as natural gas and biogas at the time of investigation and maintains the use of clean energy after energy transition.” A value of 1 indicates that the answer is “yes,” and is 0 otherwise.
3. Control variables: Many studies have revealed that population sociological factors (age, gender, marital status, region), socioeconomic status factors (education, income), health insurance factors, and lifestyle factors (whether smoking) affect people’s health status (7, 13, 21–24). Following practices established in the previous literature, we divide the variables into individual, family, and regional characteristics. The individual characteristics include gender, education, age, smoking, and health insurance; family characteristics include household size, household income, homeownership, and urban/rural area. The principal regional characteristic is north or south region. According to the traditional definition, provinces located north of the Qinling-Huaihe line are marked “north.”

We also look at three variables that effectively measure the cost imposed on households by the energy transition: the *per capita* household fuel expenditure; the energy burden; and energy-poor or not. According to previous research, the ratio of household fuel expenditure to household income is the energy burden borne by households—a measure of the severity of the energy affordability problem. Households with an energy burden higher than 10% are defined as energy-poor households (15, 18). We use these variables in the heterogeneity analysis.

The descriptive statistics of the variable above are shown in Table 1.

**Table 1:** Statistical description of primary variables.

Variables	Definition	Obs.	mean	Std.	Min.	Max.
**Dependent variable**						
Residents’ health status	Healthy = 1; otherwise = 0	34,467	0.682	0.465	0	1
**Independent variable**						
Energy transition	Yes = 1; no = 0	34,467	0.660	0.474	0	1
**Control variable**						
Education	Illiterate = 1; college degree or above = 4	34,467	2.580	0.915	1	4
Gender	Female = 1; male = 0	34,467	0.482	0.500	0	1
Age	Actual age of respondents	34,467	48.368	15.084	16	94
Smoking	Smoking = 1; non-Smoking = 0	34,467	0.275	0.446	0	1
Health insurance	Yes = 1; none = 0	34,467	0.915	0.279	0	1
Household fuel expenditure	*per capita* household fuel expenditure (take logarithm)	34,467	2.983	0.842	0	7.419
Household size	number of economically connected members in the family	34,467	4.135	1.845	1	17
Household income	*per capita* household income (take logarithm)	34,467	9.342	1.184	0.511	14.210
Homeownership	Yes = 1; no = 0	34,467	0.907	0.290	0	1
Rural	Rural = 1, Urban = 0	34,467	0.380	0.485	0	1
The North	North = 1, South = 0	34,467	0.556	0.497	0	1
Energy burden	The ratio of household fuel expenditure to total household income	34,467	0.005	0.028	0.001	1
Energy poor households	Energy burden ≥10% = 1; otherwise = 0	34,467	0.060	0.080	0	1

## 4 Results analysis

### 4.1 Descriptive statistics analysis

**Figure 1:**
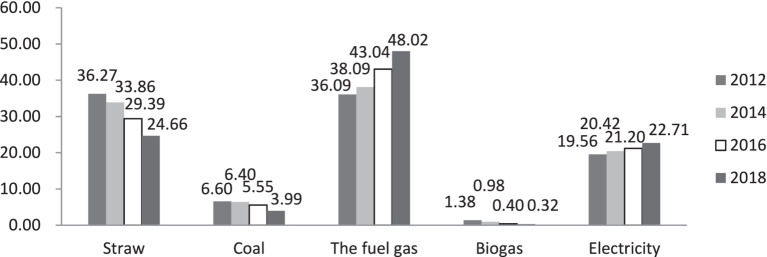
Household energy usage situation.

The energy structure of Chinese households underwent several significant changes between 2012 and 2018. Figure 1, which shows the descriptive statistics of sample energy usage, illustrates household energy use during this period. The use of firewood, coal, and biogas decreased. The percentage of households that used firewood as the main cooking fuel decreased from 36.27% in 2012 to 24.66% in 2018, while those that primarily used coal for cooking decreased from 6.60 to 3.99%. Overall, the proportion of households using solid fuels fell from 42.87% in 2012 to 28.65% in 2018. At the same time, the proportion of households that used both gas and electricity increased: gas increased from 36.09 to 48.02%, while electricity increased from 19.56 to 22.71%. In other words, households that used clean energy, such as natural gas, gas, biogas, solar energy, and electricity, increased from 57.03% in 2012 to 71.05% in 2018.

Table 2 compares the mean health status of the treated (energy transition) and control (non-energy transition) groups in the 2012 base period and the follow-up surveys. In all periods, the probability of good self-rated health in the energy transition group was higher than that in the non-transition group. That is, during the study period, the health status of the energy transition group was significantly better than that of the non-transition group.

**Table 2:** Comparison of residents’ health status before and after household energy substitution.

Dependent variable	Residents’ health status				
Year	Control Group		Treated Group		Difference
	Mean	S.D.	Mean	S.D.	
2012	0.585	0.493	0.653	0.476	0.068
2014	0.665	0.472	0.723	0.447	0.058
2016	0.591	0.492	0.664	0.473	0.073
2018	0.627	0.484	0.691	0.462	0.064

### 4.2 Health effects of the clean energy transition

In this subsection, we introduce the regression results of the DID and PSM-DID analysis. In the regression, we employ the Logit method to estimate the impact of the clean energy transition on residents’ health. The estimated results are reported in Table 3. We first show the results of the main explanatory variables (columns 1 and 4), then introduce a set of control variables (columns 2 and 5), and finally include in the model the time and province fixed effect (columns 3 and 6) to control for time and regional characteristics.

**Table 3:** Estimated results of the impact of the household energy transition on residents’ health.

	DID			PSM-DID		
	(1)	(2)	(3)	(4)	(5)	(6)
Energy transition	0.188^***^	0.269^***^	0.193^***^	0.229^***^	0.267^***^	0.193^***^
	(0.026)	(0.033)	(0.049)	(0.031)	(0.033)	(0.049)
Age		−0.039^***^	−0.039^***^		−0.038^***^	−0.039^***^
		(0.001)	(0.001)		(0.001)	(0.001)
Female		0.408^***^	0.397^***^		0.409^***^	0.398^***^
		(0.041)	(0.041)		(0.041)	(0.041)
Education		0.289^***^	0.293^***^		0.289^***^	0.292^***^
		(0.019)	(0.020)		(0.020)	(0.020)
Smoking		0.035	0.057		0.037	0.058
		(0.047)	(0.047)		(0.047)	(0.047)
Health insurance		0.031	0.017		0.032	0.019
		(0.058)	(0.059)		(0.058)	(0.059)
Household fuel expenditure		−0.047^**^	−0.036^*^		−0.048^**^	−0.036^*^
		(0.019)	(0.020)		(0.019)	(0.020)
Family size		0.027^***^	0.028^***^		0.027^***^	0.029^***^
		(0.009)	(0.010)		(0.0092)	(0.010)
Homeownership		0.139^**^	0.119^**^		0.148^***^	0.125^**^
		(0.057)	(0.058)		(0.057)	(0.058)
Rural		−0.168^***^	−0.170^***^		−0.169^***^	−0.170^***^
		(0.034)	(0.035)		(0.034)	(0.035)
The North		−0.068^**^	0.414^*^		−0.066^**^	0.4208^*^
		(0.033)	(0.228)		(0.033)	(0.228)
Constant term	1.647^***^	2.748^***^	2.409^***^	1.678***	2.734^***^	2.395^***^
	(0.020)	(0.142)	(0.211)	(0.024)	(0.143)	(0.211)
Time dummy variables	No	No	Yes	No	No	Yes
Province dummy variables	No	No	Yes	No	No	Yes
Observations	34,467	34,219	34,211	34,061	34,051	34,043
Pseudo R^2^	0.01	0.089	0.098	0.02	0.089	0.098

Figures in brackets mean standard error. ***, **, * represent the significance level of 1%, 5%, 10%, respectively, in the two-tailed test.

As shown in Table 3, the coefficients of the two methods were quite close, and all of them significantly improved the probability that residents being in good health. Indeed, the impact of the energy transition on health status was very robust. Taking the results of the PSM-DID method as an example, we see that replacing solid fuels, such as coal and firewood, with gas, biogas, and electricity during the four survey periods increased the probability of self-rated good health by about 0.193. The health effect of the energy transition was significant. This indicates that replacing solid fuels with clean energy can effectively improve the health status of residents, and Hypothesis 1 is verified.

### 4.3 Robustness check

#### 4.3.1 Dependent variable substitution

To ensure the robustness of the regression results, we replace the dependent variable with the following dummy variables: unhealthy, which indicates that the respondent felt unwell during the past 2 weeks; chronic disease, which indicates that the respondent had a chronic disease during the past 6 months; and hospitalized, indicating the respondent was hospitalized during the past year. The DID method is used for regression, and the results are shown in Table 4. Although in some models the significance of the key independent variable (the energy transition) decreased, the meaning represented by the coefficient symbols was consistent with the findings presented above; that is, the clean energy transition decreased residents’ health risk and improves their health status. These results support the robustness of the benchmark regression conclusion.

**Table 4:** Estimated results of replacing dependent variables.

	Self-rated health	Unhealthy	Chronic diseases	Hospitalized
	(1)	(2)	(3)	(4)
Energy transition	0.193^***^	−0.161^***^	−0.035^*^	−0.039
	(0.049)	(0.038)	(0.046)	(0.053)
Constant	1.678^***^	−1.008^***^	−4.206^***^	−4.118^***^
	(0.024)	(0.156)	(0.213)	(0.233)
Control variables	Yes	Yes	Yes	Yes
Time dummies	Yes	Yes	Yes	Yes
Province dummies	Yes	Yes	Yes	Yes
Observations	34,466	34,440	34,413	34,438
Pseudo R^2^	0.094	0.030	0.081	0.065

Figures in brackets mean standard error. ***, **, * represent the significance level of 1%, 5%, 10%, respectively, in the two-tailed test.

#### 4.3.2 Placebo test

**Figure 2:**
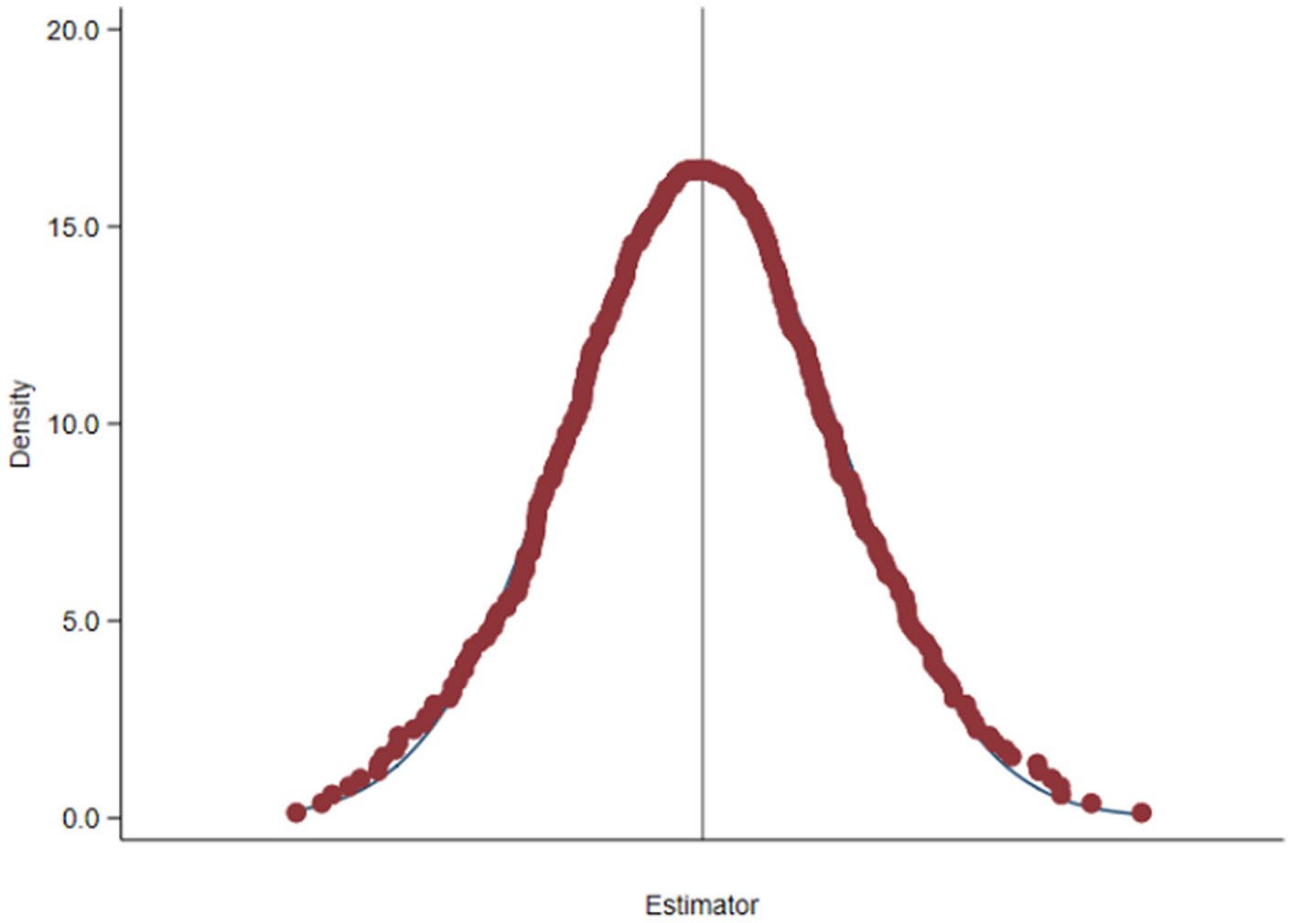
Nuclear density distribution.

Health status is affected by multiple factors. To control for heterogeneity due to missing variables or changes over time, the placebo test is designed, referring to an existing research practice (13, 15). The treated group and control group remain unchanged. The Bootstrap method is used for placebo testing, and the model in formular (5) is repeatedly estimated. If the estimated coefficient of the explanatory variable is significantly different from 0, the model may have identification bias. Figure 2 shows the estimated coefficients and T-values of the randomly generated treated group and the control group, and the distribution of the estimated coefficients was significantly close to 0, which confirmed the robustness of the conclusion.

#### 4.3.3 Parallel trend test

To determine whether the completion of the household energy transition has had a positive impact on the health status of residents, we carry out a parallel trend test. Based on the survey data from 2012, 2014, 2016, and 2018, we divide the sample interval into six segments from 6 years before the energy transition to 6 years after the transition. Thus, we construct six indicator variables: yb3 (6 years before the transition), yb2, yb1, ya1 (2 years after the transition), ya2, and ya3. When the household is in the subsample interval, the indicator variable is 1; otherwise, it is 0.

**Figure 3:**
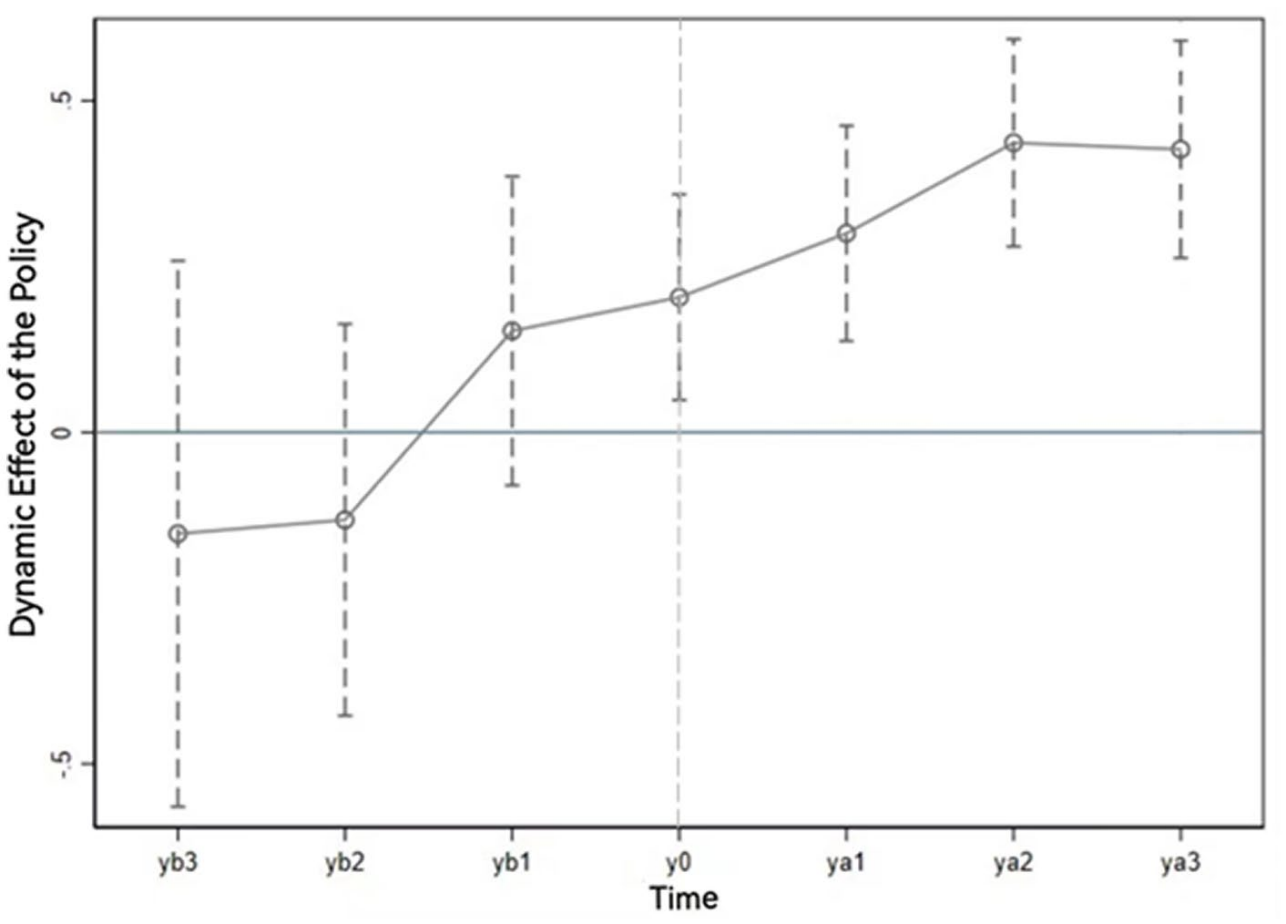
Dynamic effect diagram.

Figure 3 shows the results of the dynamic heterogeneity analysis. If the confidence interval of a dummy variable includes 0 points, then the coefficient of the dummy variable is not significant. If the confidence interval is above 0, then the coefficient is significantly positive. As shown in Figure 3, the coefficients of yb3, yb2, and yb1 were not significant, indicating that there was no significant difference in health status between the treated group and the control group prior to the transition in household energy consumption. Thus, the parallel trend hypothesis is established. After the energy transition, the estimated coefficients of each dummy variable were significantly positive at the level of 5%, indicating that the use of clean energy significantly improved the health level of residents. From the perspective of dynamic heterogeneity, in the second to fourth years after the energy transition, the residents’ health level showed a gradual increasing trend, and after 4 years, the long-term impact of clean energy transition on residents’ health tended to became stable, indicating that the energy transition had a dynamic and sustainable effect on residents’ health.

### 4.4 Heterogeneity analysis

According to the overall analysis results, a household’s clean energy transition is expected to affect the health of family members. Thus, we ask: Do different groups of residents obtain the same health benefits? In this paper, residents are grouped and tested based on gender, age, household income level, energy burden, and region. The DID results are shown in Table 5.

**Table 5:** Health effects of the energy transition on different characteristic groups.

Characteristics	Categories	Coef.	Obs.	Control vari.	Time dummy	Province dummy	Pseudo R^2^
Gender	Female	0.267^***^ (0.064)	18,166	Yes	Yes	Yes	0.103
	Male	0.172^***^ (0.0740)	15,759	Yes	Yes	Yes	0.075
Age	Old people	0.331^***^ (0.095)	6,047	Yes	Yes	Yes	0.034
	Young and middle-aged	0.182^***^ (0.067)	25,900	Yes	Yes	Yes	0.092
Income	Low-income	0.095 (0.083)	7,715	Yes	Yes	Yes	0.095
	Non-low-income	0.238^**^ (0.100)	26,654	Yes	Yes	Yes	0.086
Energy burden	Energy poverty	0.338 (0.261)	1,675	Yes	Yes	Yes	0.126
	Non-energy poverty	0.190^***^ (0.050)	32,940	Yes	Yes	Yes	0.094
Region	Rural	0.195^***^ (0.071)	11,250	Yes	Yes	Yes	0.094
	Urban	0.294^***^ (0.084)	19,707	Yes	Yes	Yes	0.081

Figures in brackets mean standard error. ***, **, * represent the significance level of 1%, 5%, 10%, respectively, in the two-tailed test.

The regression results of gender grouping show that after the improvement in fuel structure, women’s health improved significantly more than men’s health (with the coefficient 0.267 vs. 0.172). Consistent with the realistic analysis, because women did most of the cooking, the transition to clean energy and reductions in solid fuel use particularly reduced women’s health risks. When residents are grouped according to age characteristics, the regression results show that the clean energy transition increased the health status of older adults disproportionately. For older adult residents aged 60 and above, the energy transition had health effects that significantly increased the probability of self-rated good health. That is, older adult residents, too, had a higher return on health from clean energy substitution. As noted previously, women and older people generally have a lower socioeconomic status and suffer more severe environmental health hazards than men and younger people. The clean energy transition can lead to improvements in their health status. Thus, we infer that life energy substitution will help promote health equity, thus verifying hypothesis 2.

Next, we compare low-income (according to the local low-income division criteria) and non-low-income residents. The DID results show that the clean energy transition significantly improved the health status of non-low-income residents, while in the low-income group, the health effects were not significant. Moreover, the coefficient of the non-low-income group was much higher than that of the low-income group. Because the above method cannot directly determine whether the coefficient difference between these two groups is significant, we further added the interaction term Energy transition × Low-income. The results show that the coefficient of this interaction term was statistically significant (coefficient = −0.04, *p* = 0.003), indicating that the impact of the energy transition on health status was greater in non-low-income groups.

The health outcomes of the energy transition differed significantly in energy-poor and non-energy-poor groups. In the non-energy-poor group, the estimated coefficient was significantly positive at the 1% confidence interval, but in the energy-poor household sample, it was negative. Although the estimated coefficient was not significant, it indicated that the energy transition negatively impacted the health status of energy-poor residents.

Not surprisingly, the energy transition had strikingly different effects in rural and urban groups. The results indicated that the health effects of the energy transition were greater among urban residents, although these effects were significant for residents in both urban and rural areas. Furthermore, when we added the interaction term of energy transition and urban/rural area, we found that the coefficient of the interaction term was statistically significant, verifying that, in terms of health status, urban residents were the primary beneficiaries of the clean energy transition.

Generally, non-clean-energy-dependent households use fuelwood and other traditional solid fuel to meet their daily energy needs, and most solid fuels are available for free or at a very low price. When users in economically poor or energy-poor households (most of which are in rural areas) turn to modern energy sources, they often find that, because their access to clean energy is poor, the clean energy is costly. In these households, the energy transition can lead to a deterioration in energy affordability, creating an urgent economic burden for them. The extra money they spend on energy can crowd out expenditures on other consumables, such as food and healthcare. The higher the proportion of energy expenditure to household income, the more severe the crowding out of other consumption expenditures. This squeezed-out consumption can affect residents’ health status, particularly in energy-poor households. It can also mask the positive health effects of the energy transition. This confirms Hypothesis 3 and suggests that the clean energy transition exacerbates health inequalities.

## 5 Conclusions and policy recommendations

Protecting the air environment allows people to pursue a better life. To improve air quality, the Chinese government is actively promoting replacing high-polluting energy sources, such as coal, with electricity and gas in the production and household sectors. The policy goal is to reduce the health risks caused by air pollution. Based on the four waves of CFPS survey data from 2012 to 2018, this paper uses the DID regression method to investigate the impact of the clean energy transition on the health status of residents. Extrapolating from our findings we reach four conclusions.

1. Generally speaking, the use of cleaner cooking fuels significantly improves residents’ health status. This conclusion is robust under a variety of conditions, indicating that in China, improvements in fuel structure can lead to improvements in residents’ health. This has great significance for the overall goal of building a prosperous society and accelerating socialist modernization.
2. Heterogeneity test results show that groups with relatively lower socioeconomic status, including the older adult and women, enjoy significantly improved health returns from the clean energy transition. These results hold when a variety of methods are applied to the whole sample, including when it is subdivided by age and gender. If the energy transition improves the health of older people and women, it would contribute to improving residents’ overall well-being and health equity.
3. Heterogeneity test results also show that the clean energy transition has the greatest health impact on residents who can easily obtain clean energy or replace non-clean energy at an affordable cost. In contrast, rural residents in non-clean-energy-dependent households often have lower-than-average incomes and, thus, experience energy affordability and availability problems. In these households, the health returns from the energy transition can be low or insignificant. In some households, in other words, the clean energy transition exacerbates health inequities.

These findings provide empirical evidence of the effects of the energy transition on health, and, highlighting the social significance of promoting clean energy, they provide an important reference for the realization of the health of all people through the construction of a healthy environment and a healthy China. According to these findings, in the construction of a healthy environment and the implementation of a healthy China strategy, the government can adopt the following countermeasures:

First, upgrading the energy structure should be linked to constructing a healthy China. The environmental hazards and health risks of traditional fuels should be emphasized to stimulate residents’ consciousness and encourage energy substitution.

Second, the government should increase spending on energy upgrading in rural areas and emphasize the role that energy plays in human health in both urban and rural areas. Rural residents who are poor and have a low level of education level as well as the older adult are more vulnerable because of their limited ability to purchase clean energy. Because they remain largely dependent on traditional solid fuels, they suffer more health risks and are more likely to be pushed into the poverty trap by energy structure problems. These circumstances could be relieved by providing poor and low-income rural residents with subsidies to purchase cleaner energy, such as natural gas and electricity. Authorities should take into account the cost of clean energy use when they set the minimum protection line.

Third, because energy poverty weakens the health effects of the clean energy transition, eliminating energy poverty is an important issue in China’s anti-poverty strategy in the new era. Initiatives to alleviate and eliminate energy poverty should consider the affordability and availability of energy consumption in poor groups. To this end, the construction of energy infrastructure in backward urban and rural areas should be accelerated, and energy consumption subsidies for poor groups should be increased. These steps will allow more residents to use clean energy more easily in their lives.

## Data availability statement

Publicly available datasets were analyzed in this study. This data can be found at: http://www.isss.pku.edu.cn/cfps/index.htm.

## Author contributions

LW: Funding acquisition, Writing – review & editing. QL: Writing – original draft. LL: Writing – original draft, Data curation.
